# Does the socioeconomic status predict health service utilization in persons with enhanced health care needs? Results from a population-based survey in persons with spinal cord lesions from Switzerland

**DOI:** 10.1186/s12939-022-01693-6

**Published:** 2022-07-12

**Authors:** Christine Fekete, Caroline Debnar, Anke Scheel-Sailer, Armin Gemperli

**Affiliations:** 1 Swiss Paraplegic Research, Guido A. Zäch Strasse 4, 6207 Nottwil, Switzerland; 2 Department of Health Sciences & Medicine, University of Lucerne, Frohburgstrasse 2, 6003 Lucerne, Switzerland; 3 Work Mastery, Corporate Health Consulting, Pilatusstrasse 19, 6003 Lucerne, Switzerland; 4 Swiss Paraplegic Center, Guido A. Zäch Strasse 1, 6207 Nottwil, Switzerland; 5 Center of Primary and Community Care, University of Lucerne, Frohburgstrasse 2, 6003 Lucerne, Switzerland

**Keywords:** Socioeconomic status, Social inequalities, Health service utilization, Spinal cord injury, Switzerland

## Abstract

**Background:**

Evidence suggests that the socioeconomic status (SES) affects individuals’ health service utilization. Spinal cord injury is a condition that often leads to physical impairments and enhanced health care needs. It therefore presents an informative and yet under-researched case in point to investigate social inequalities in health service utilization. This study aims to describe associations between SES and health service utilization in adults with spinal cord injury from Switzerland.

**Methods:**

We use cross-sectional data from 1,294 participants of the Swiss Spinal Cord Injury Cohort Study community survey 2017. SES was operationalized with education, household income, perceived financial hardship, subjective status, and granting of supplementary financial benefits. Health service utilization was assessed with information on visits to 13 different health care providers and four health care institutions (inpatient stays, outpatient clinics, emergency departments, specialized spinal cord centers) during the past 12 months. The dichotomized outcomes on service utilization (visited vs. not visited) were regressed on SES indicators, including adjustments for sociodemographics, lesion characteristics, and health status.

**Results:**

Persons with higher SES reported higher likelihood for specialist, dentist, and dental hygienist visits and reported utilizing a larger number of different care providers. Further, specific SES indicators were associated with certain care provider visits (i.e., higher education and subjective status: higher odds for pharmacist visits; higher income: higher odds for natural healer visits; higher subjective status: higher odds for chiropractor visits; supplementary benefit granting: higher odds for general practitioner and home care service visits). We found statistically non-significant trends towards lower likelihood for inpatient stays, outpatient clinic and emergency department visits and enhanced likelihood for specialized spinal cord-center visits in higher SES groups.

**Conclusions:**

This study generally supports the claim that basic health care provision is guaranteed for all patients with spinal cord injury in Switzerland, independently of their SES. However, social inequalities were still observed for the utilization of specific providers, such as oral health care providers. Given that oral health is key for health maintenance in persons with spinal cord injury, specific interventions to enhance regular dental check-ups in lower SES groups are highly recommended.

**Supplementary Information:**

The online version contains supplementary material available at 10.1186/s12939-022-01693-6.

## Background

Equity in access to health care services is an important policy objective and a key indicator of health system performance in OECD countries, proclaiming equal access to health care for everyone based on their needs, irrespective of their socioeconomic status (SES) [1, 2]. Despite this claim, previous research demonstrated that health service utilization is not equally distributed across different SES groups. A study using data from 18 selected OECD countries observed for nearly all countries that persons with higher income more often consulted specialists, more often visited dentists, and reported more cancer screenings than persons with lower incomes, even after adjustment for differences in health care needs [3]. Likewise, a systematic review of 26 studies from countries with universal health coverage confirmed that specialist hospital services were more often used by persons with higher income, taking into account differential health needs across SES groups [4]. Similar findings were reported for the SES indicator occupational status, as a study based on six waves of the Spanish National Health Survey showed that persons with non-manual occupations were more likely to visit specialized services and dentists, but were less likely to visit general practitioners and emergency departments than persons with manual occupations [5].

Despite the fact that Switzerland is a high-income country with universal health coverage and a highly developed social welfare system, social inequalities in health service utilization were also observed for the Swiss context [6–12]. Studies report different patterns of utilization across SES groups [6–8, 10–12], but also provide evidence for income inequalities in unmet health care needs [9]. Available studies are however restricted to specific SES indicators, such as the granting of social benefits, education and/or income, or and did not adjust findings for social inequalities in health care needs [6]. As it is widely recognized that the different SES indicators assess different dimensions related to distinct health-related resources and are oftentimes inconsistently or weakly to moderately correlated within individuals [13], it is important to study different SES indicators separately to disentangle underlying mechanisms linking them to service utilization patterns. Of note, this study is solely focused on the investigation of socioeconomic differences in health service utilization and not so on other dimensions of inequalities, such as gender, race, ethnicity or age.

In this study, we aim to contribute to current evidence on socioeconomic inequalities in health service utilization in Switzerland, focusing on persons with enhanced health care needs, namely persons with spinal cord injury (SCI). Persons with SCI sustain a complete or partial loss of sensory and motor function below the lesion level, putting constraints on their health and functioning, which ultimately considerably increases their need for health care. For the SCI population in Switzerland, several studies already detailed on health service utilization [14–17], but associations with SES have not been investigated with the exception of one study documenting more general practitioner visits in lower income groups [16]. However, those results were not adjusted for different health care needs across SES groups. The objective of the present study is to describe social inequalities in health services utilization in a population-based sample of persons with SCI in Switzerland. More specifically, we aim to examine associations of the SES indicators education, household income, perceived financial hardship, subjective social status, and granting of supplementary benefits with visits to 13 specific health care providers and four health care institutions.

## Methods

### Design and participants

We used cross-sectional data from the second population-based community survey of the Swiss Spinal Cord Injury Cohort Study (SwiSCI), including community-dwelling Swiss residents aged over 16 years with traumatic or non-traumatic SCI [18]. Persons with congenital conditions leading to SCI, neurodegenerative disorders, and Guillain-Barré syndrome were excluded from the study. Participants were recruited based on databases from the Swiss Paraplegic Association (organization representing people with SCI), ParaHelp (SCI home care service) and the four specialized SCI-centers in Switzerland. Recruitment and data collection were performed between March 2017 and March 2018 and the survey included two questionnaires that were sent to participants with an interval of 4–6 weeks. The invited source population included 3,959 eligible persons, whereby 1,294 persons completed both questionnaires (response rate 32.7%) [18]. Participants were offered paper-and-pencil or online questionnaires, or telephone interviews and the questionnaires were available in the tree official Swiss languages (German, French, Italian). Further details on the design of the SwiSCI cohort study, the recruitment outcomes, participation rates, and non-response bias in the community survey 2017 can be found elsewhere [18–20].

### Measures

#### Socioeconomic status

Besides the traditional SES indicators education and household income, we also included more subjective SES indicators perception of financial hardship and subjective social status as well as information on whether people are granted supplementary financial benefits as an objective indicator of enhanced poverty risk. Education was operationalized with information on the highest educational attainment and was recoded into the four categories primary or lower secondary; upper secondary; short-term tertiary; and middle or long-term tertiary education. The mean reported household income was matched to the income distribution in deciles at the Swiss population level to describe a persons’ relative income position weighted by the household size, as described elsewhere in detail [21]. Financial hardship was evaluated with an item from the Nottwil Environmental Factors Inventory [22] on the impact of problematic financial situations on participants life during the past month (not applicable, no influence, made my life a little harder, made my life a lot harder). A 3-categorical variable was used for analysis, including the categories ‘not applicable or none’, ‘some’, and ‘massive financial hardship’. Financial hardship has previously been associated with different health indicators in persons with SCI in Switzerland [23, 24]. The MacArthur Scale of Subjective Social Status was used to measure the subjective perception of one’s social status represented by a 10-rung ladder [25], which has been repeatedly associated with health indicators in general population samples [26, 27]. Participants crossed the rung on which they would place themselves and higher values indicate a higher subjective social status. In Switzerland, the supplementary benefits assist people who cannot cover the minimum living costs with other forms of income (e.g. from paid work, disability or old age pension). Information on the granting of supplementary benefits is gathered with an item asking participants about the composition of their current income, whereby those who indicated that supplementary benefits are part of their income were coded with ‘supplementary benefits’ and others with 'no supplementary benefits’.

#### Health service utilization

##### Visits to health care providers

Information on the visit of 15 different health care providers during the past 12 months were assessed as dichotomous variables (visited vs. not visited): general practitioner; SCI-specialist; other specialist; dentist; dental-hygienist; psychologist; occupational therapist; physiotherapist; chiropractor; masseur; natural healer; pharmacist; Spitex (Swiss home care service), speech therapist and midwife. Information on the visits of speech therapists and midwives were excluded from analysis due to low prevalence of utilization (1.2% and 5.0% of the sample, respectively). We created a variable indicating the number of different health care providers visited during the past 12 months by summing up all the visited providers included in analysis (range 0–13). The item listing the 15 health care providers also included a free text response to list additional health care providers and a response option ‘no health care provider visited’. The low number of persons that indicated not having visited any health care provider in the past 12 months (*n* = 11) precluded us from studying associations between not having visited health care providers and SES indicators. Persons who did not check the box ‘no health care provider visited’, did not check any of the health care providers, and left the free text response blank, were assigned a missing value.

##### Visits to health care institutions

Information on inpatient stays, visits to outpatient clinics, unplanned visits to emergency departments, and visits to specialized paraplegic centers during the past 12 months were used to assess the use of health care services in the past 12 months (dichotomous, ‘yes’ vs. ‘no’).

#### Covariates

Age, gender, SCI severity (incomplete paraplegia; complete paraplegia; incomplete tetraplegia; complete tetraplegia), time since injury, and car driving time to closest SCI-center in minutes were used as potential confounders. Given that persons from lower SES have a higher disease burden, adjusting for differences in health service needs is an important prerequisite to adequately reflect SES-patterns in health service utilization. We therefore include secondary health conditions, mental health and comorbidities as indicators for health status to be included as covariates. Mental health was assessed with the 5-item SF-36 Mental Health Index (MHI-5, version 2) [28], assessing the frequency of emotional states in the past four weeks on a 5-point scale (0 = all of the time to 4 = none of the time). The raw score was transformed to a 0–100 scale according to established guidelines [28]. Secondary health conditions were measured using the Spinal Cord Injury Secondary Conditions Scale (SCI-SCS) [29]. The SCI-SCS assessed the burden of 14 secondary health conditions that are commonly diagnosed in persons with SCI over the past three months on a 4-point scale (0 ‘not existing or insignificant’; 1 ‘mild or infrequent’, 2 ‘moderate or occasional’, 3 ‘severe or chronic’). A sum score ranging from 0–42 was built for analysis. Comorbidities were assessed with three dichotomous items on the presence or absence of cancer, coronary heart disease, and diabetes from the Self-Administered Comorbidity Questionnaire [30].

### Statistical analysis

All analyses were conducted using STATA Version 16.0 for Windows (College Station, TX, USA). We first describe the distribution of variables of interest. Second, logistic regressions were used to explore associations between SES and health service utilization. We report odds ratios (OR) and 95% confidence intervals (CI) and respective *p*-values from global tests. Two subsequent models were run: 1) unadjusted, 2) adjusted for the covariates age, gender, SCI severity, time since injury, driving time to closest SCI-center, and health status including the indicators secondary health conditions, mental health, and comorbidities. The continuous variables income deciles (range 1–10) and subjective social status (range 1–10) were used as categorical variables in sensitivity analysis using four groups to identify potentially non-linear trends. Statistically significant linear trends were largely confirmed in the analysis using categorical predictors (results not shown).

To assess potential bias due to missing values, analyses were repeated with complete and imputed data in sensitivity analysis. Missing values were imputed with multiple imputation (MI) by chained equations on 20 imputed datasets [31], assuming that data were missing at random. Descriptive results are shown based on complete data, results from regression modelling are based on imputed data.

## Results

Table 1 shows basic characteristics of the study population. Participants were predominantly male (71.1%), with mean age of 56.4 years. Incomplete paraplegia was the most frequent (42.6%) and complete tetraplegia the least frequent SCI severity (7.7%). On average, participants lived 18.8 years with SCI. The average car driving time to a specialized SCI-center was around 38 min. On average, participants scored 12.5 on the secondary health conditions scale (range 0–42), 73.3 on the mental health scale (range 0–100) and indicated on average 0.2 comorbidities (range 0–3). Around 17.3% of the sample reported lower secondary, 44.5% higher or post-secondary, 18.3% short tertiary and 20.0% tertiary education as highest educational level. Participants scored their subjective status on average with 5.6 on the 1–10 scale, 7.3% indicated experiencing financial hardship and 7.9% of the sample was granted supplementary benefits.

**Table 1:** Description of the SwiSCI study population 2017

Variables [% of missing values in total sample]	Total (*n* = 1,294)	
	N (%)	Mean (SD); median (IQR)
**Demographic characteristics**		
Male gender [0]	920 (71.1)	
Age in years [0]		56.4 (14.4); 57 (46–67)
Distance to closest SCI-center (min by car) [1.6]		38.1 (21.0); 35 (22–52)
**Lesion characteristics**		
Years since injury [6.1]		18.8 (13.1); 15.8 (7.7–27.5)
Severity of the spinal cord injury [9.3]		
Incomplete paraplegia	500 (42.6)	
Complete paraplegia	324 (27.6)	
Incomplete tetraplegia	260 (22.2)	
Complete tetraplegia	90 (7.7)	
**Health status**		
Secondary conditions, range 0–42 [25.3]		12.5 (6.9); 12 (7–17)
Mental health, range 0–100 [5.1]		73.3 (17.7); 78 (62–88)
Comorbidities, range 0–3 [3.2]		0.2 (0.5); 0 (0–0)
**Socioeconomic status**		
Highest education [3.2]		
Compulsory	217 (17.3)	
Upper or post-secondary	557 (44.5)	
Short tertiary	229 (18.3)	
Tertiary	250 (20.0)	
Household income deciles, range 1–10 [23.4]		4.3 (2.7); 4 (2–6)
Financial hardship [3.0]		
None	962 (76.7)	
Some	201 (16.0)	
Massive	92 (7.3)	
Subjective social status, range 1–10 [4.7]		5.6 (1.9); 6 (4–7)
Granted supplementary benefits [0]	102 (7.9)	

*Abbreviations: IQR* Interquartile Range, *SCI* Spinal Cord Injury, *SD* Standard Deviation

**Fig. 1:**
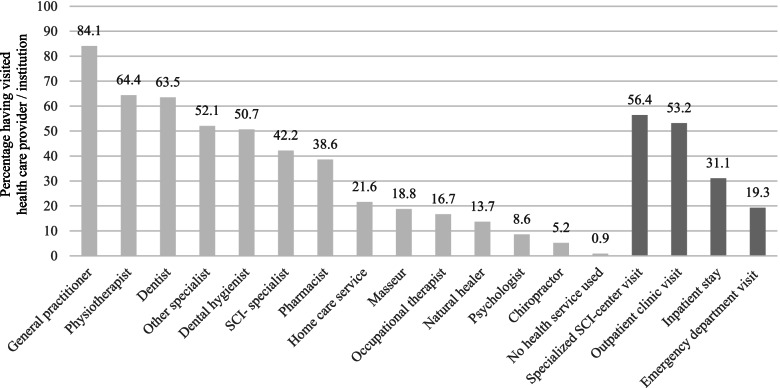
Percentage of persons having visited respective health care providers (light grey) or institutions (darker grey) during the past 12 months

Figure 1 displays the proportion of persons having visited the specific health care providers and institutions during the past 12 months. A large majority of the sample visited general practitioners, followed by physiotherapists, dentists, specialists, and dental hygienists. Around four out of 10 persons visited SCI-specialists and pharmacists, and about one fifth of the sample indicated visits by home care services or having visited masseurs. Occupational therapists, natural healers, psychologists, and chiropractors were visited by a smaller proportion of the sample. Only one out of hundred participants indicated having not visited any health care provider during the past 12 months. Over half of the sample visited a specialized SCI-center or an outpatient clinic, almost one third of the sample reported an inpatient stay, and one fifth indicated having visited an emergency department during the past 12 months.

### Socioeconomic status and visits to different health care providers

Table 2 shows adjusted associations of the SES indicators with visits to 13 different health care providers and the total number of visited care providers (unadjusted associations can be found in Additional file 1). We observe positive associations between education and visits to SCI- and other specialists, dentists, dental hygienists, and pharmacists. Increased household income was related to more visits to dentists, dental hygienists, and natural healers. Persons with no or only some financial hardship reported more visits to dental hygienists as compared to those with massive financial hardship. Persons who were granted supplementary benefits were more likely to visit general practitioners and to receive support from a home care service, but were less likely to visit SCI-specialists and dental hygienists than persons not being granted supplementary benefits. Higher subjective social status was associated with higher likelihood to visit SCI-specialists, dentists, dental hygienists, chiropractors, and pharmacists. Visits to physiotherapists, occupational therapists, masseurs and psychologists were not related to any of the SES indicators. Overall, results indicate that persons with higher SES tend to visit more different health care providers (*p* < 0.05 for education, income, and subjective social status). Results from full case analyses and results from imputed datasets were compared and no relevant differences between the two strategies were detected.

**Table 2:** Adjusted associations between indicators of the socioeconomic status and visits to specific health care providers: Odds ratio (OR) and 95% confidence intervals (CI) for the likelihood to visit specific providers and coefficients (coeff) and 95% CI for the total number of providers visited during the past 12 months by indicators of the socioeconomic status

	**General practitioner**	**SCI-specialist**	**Other specialists**	**Dentist**	**Dental hygienist**	**Physio-therapist**	**Occupational therapist**	**Chiropractor**	**Home care service**	**Masseur**	**Pharmacist**	**Natural healer**	**Psychologist**	**N° of different health care providers visited (range 0–13)**
	OR (95% CI)	OR (95% CI)	OR (95% CI)	OR (95% CI)	OR (95% CI)	OR (95% CI)	OR (95% CI)	OR (95% CI)	OR (95% CI)	OR (95% CI)	OR (95% CI)	OR (95% CI)	OR (95% CI)	Coeff (95% CI)
**Highest education**														
Compulsory	Ref	Ref	Ref	Ref	Ref	Ref	Ref	Ref	Ref	Ref	Ref	Ref	Ref	Ref
Higher secondary	1.09 (0.67–1.77)	**1.22 (0.87–1.72)**	**1.61 (1.14–2.27)**	**1.20 (0.85–1.70)**	**2.12 (1.51–2.97)**	1.27 (0.89–1.80)	1.25 (0.79–1.98)	1.58 (0.66–3.76)	1.06 (0.71–1.59)	1.41 (0.90–2.20)	**1.40 (0.99–1.99)**	1.44 (0.86–2.42)	0.99 (0.54–1.81)	**0.64 (0.32–0.97)**
Short tertiary	1.63 (0.89–2.99)	**1.43 (0.96–2.15)**	**2.07 (1.37–3.13)**	**1.61 (1.07–2.43)**	**2.89 (1.93–4.32)**	1.18 (0.78–1.77)	1.11 (0.64–1.92)	2.92 (1.15–7.40)	1.05 (0.64–1.73)	2.25 (1.36–3.72)	**1.43 (0.94–2.18)**	1.95 (1.08–3.51)	1.20 (0.59–2.45)	**1.07 (0.68–1.46)**
Tertiary	0.78 (0.46–1.31)	**1.77 (1.19–2.61)**	**2.31 (1.54–3.45)**	**1.65 (1.10–1.47)**	**3.23 (2.19–4.78)**	1.32 (0.88–1.97)	0.99 (0.57–1.70)	2.04 (0.79–5.27)	0.93 (0.57–1.50)	1.40 (0.85–2.33)	**2.01 (1.36–2.99)**	1.72 (0.98–3.04)	1.25 (0.64–2.44)	**1.04 (0.66–1.41)**
*p-value*	*0.264*	*0.012*	< *0.001*	*0.045*	< *0.001*	*0.329*	*0.444*	*0.324*	*0.798*	*0.295*	*0.002*	*0.168*	*0.682*	< *0.001*
**Household income**														
Income deciles (1–10)	0.97 (0.91–1.03)	1.05 (0.99–1.10)	1.03 (0.98–1.09)	**1.05 (1.00–1.10)**	**1.14 (1.08–1.19)**	1.00 (0.95–1.05)	1.00 (0.93–1.07)	1.02 (0.92–1.14)	0.99 (0.93–1.05)	1.04 (0.98–1.10)	1.03 (0.98–1.08)	**1.07 (1.00–1.14)**	1.00 (0.92–1.09)	**0.05 (0.00–0.10)**
*p-value*	*0.301*	*0.086*	*0.186*	*0.033*	< *0.001*	*0.914*	*0.910*	*0.647*	*0.759*	*0.208*	*0.285*	*0.049*	*0.933*	*0.033*
**Financial hardship**														
Massive	Ref	Ref	Ref	Ref	Ref	Ref	Ref	Ref	Ref	Ref	Ref	Ref	Ref	Ref
Some	1.88 (0.93–3.80)	1.19 (0.70–2.02)	0.77 (0.45–1.33)	1.14 (0.68–1.91)	**1.66 (0.98–2.81)**	1.50 (0.87–2.58)	1.66 (0.85–3.21)	1.05 (0.32–3.52)	1.57 (0.82–3.00)	0.87 (0.48–1.58)	0.92 (0.54–1.55)	1.05 (0.48–2.29)	0.54 (0.25–1.16)	0.34 (-0.19–0.86)
None	1.93 (1.03–3.60)	1.19 (0.74–1.91)	0.80 (0.49–1.31)	1.55 (0.97–2.49)	**2.39 (1.48–3.84)**	1.55 (0.95–2.52)	1.10 (0.59–2.02)	1.15 (0.39–3.42)	1.26 (0.69–2.30)	0.73 (0.43–1.25)	1.04 (0.65–1.67)	1.41 (0.70–2.84)	0.66 (0.34–1.26)	0.48 (0.01–0.95)
*p-value*	*0.112*	*0.766*	*0.626*	*0.051*	< *0.001*	*0.214*	*0.104*	*0.946*	*0.336*	*0.412*	*0.741*	*0.356*	*0.280*	*0.114*
**Supplementary benefits**														
Yes	Ref	Ref	Ref	Ref	Ref	Ref	Ref	Ref	Ref	Ref	Ref	Ref	Ref	Ref
No	**0.34 (0.15–0.80)**	**1.80 (1.15–2.83)**	0.88 (0.57–1.37)	0.98 (0.63–1.52)	**1.68 (1.09–2.57)**	1.12 (0.72–1.76)	1.13 (0.64–1.98)	2.04 (0.62–6.76)	**0.57 (0.35–0.92**)	1.18 (0.69–2.03)	1.00 (0.65–1.54)	1.87 (0.93–3.75)	0.74 (0.39–1.39)	0.16 (-0.26–0.58)
*p-value*	*0.014*	*0.011*	*0.572*	*0.937*	*0.018*	*0.616*	*0.682*	*0.243*	*0.022*	*0.549*	*0.991*	*0.079*	*0.346*	*0.455*
**Subjective social status**														
Range 1–10	0.97 (0.89–1.06)	**1.09 (1.02–1.17)**	1.02 (0.96–1.09)	**1.14 (1.06–1.21)**	**1.20 (1.12–1.28)**	1.00 (0.94–1.08)	0.93 (0.86–1.02)	**1.18 (1.02–1.36)**	0.99 (0.91–1.07)	1.08 (1.00–1.17)	**1.08 (1.01–1.16)**	1.09 (1.00–1.20)	0.98 (0.87–1.10)	**0.13 (0.07–0.19**)
*p-value*	*0.555*	*0.007*	*0.531*	< *0.001*	< *0.001*	*0.844*	*0.108*	*0.022*	*0.728*	*0.051*	*0.017*	*0.061*	*0.729*	< *0.001*

Results based on imputed data (*n* = 1,294). Missing values in % of total sample for health care providers: 2.1%. **Results in bold** indicate statistically significant associations (*p* < 0.05)

Models adjusted for age, gender, severity of the spinal cord injury, time since injury, driving time to closest SCI-center, secondary health conditions, mental health, and comorbidities

*Abbreviations: Ref* Reference group, *SCI* Spinal Cord Injury

### Socioeconomic status and visits to different health care institutions

Table 3 shows unadjusted and adjusted associations between the different SES indicators and visits to four health care institutions. We observe a tendency towards lower likelihood for inpatient stays, outpatient clinic, and emergency department visits, and a slightly enhanced likelihood to visit a specialized SCI-center in persons with higher SES. However, besides the decreased odds to visit an outpatient clinic in persons without supplementary benefits as compared to those with supplementary benefits, associations were above the conventional level of statistical significance (p>0.05) for all SES indicators after adjustment of potential confounders.

**Table 3:** Unadjusted and adjusted associations between indicators of the socioeconomic status and inpatient clinic, outpatient clinic visits: Odds ratio (OR) and 95% confidence intervals (CI) from logistic regressions

	**Inpatient stay**		**Outpatient clinic visit**		**Visit to specialized SCI-center**		**Emergency department visit**	
	Model 1	Model 2	Model 1	Model 2	Model 1	Model 2	Model 1	Model 2
	OR (95% CI)	OR (95% CI)	OR (95% CI)	OR (95% CI)	OR (95% CI)	OR (95% CI)	OR (95% CI)	OR (95% CI)
**Highest education**								
Compulsory	Ref	Ref	Ref	Ref	Ref	Ref	Ref	Ref
Higher secondary	0.78 (0.56–1.09)	0.85 (0.60–1.21)	0.91 (0.66–1.26)	0.95 (0.68–1.33)	1.25 (0.91–1.71)	1.15 (0.83–1.61)	0.74 (0.50–1.08)	0.80 (0.54–1.20)
Short tertiary	0.88 (0.60–1.30)	1.08 (0.71–1.64)	1.02 (0.69–1.49)	1.09 (0.73–1.63)	1.37 (0.93–2.01)	1.27 (0.85–1.89)	1.03 (0.66–1.60)	1.14 (0.72–1.82)
Tertiary	0.62 (0.41–0.92)	0.72 (0.47–1.09)	0.96 (0.66–1.40)	0.97 (0.66–1.45)	1.18 (0.82–1.71)	1.10 (0.75–1.62)	0.68 (0.43–1.09)	0.74 (0.46–1.20)
*p-value*	*0.059*	*0.295*	*0.844*	*0.959*	*0.395*	*0.697*	*0.200*	*0.429*
**Household income**								
Income deciles 1–10	0.97 (0.92–1.02)	0.98 (0.93–1.03)	0.97 (0.93–1.01)	0.98 (0.94–1.02)	1.04 (1.00–1.09)	1.04 (0.99–1.09)	0.97 (0.92–1.03)	0.99 (0.93–1.05)
*p-value*	*0.260*	*0.488*	*0.139*	*0.325*	*0.080*	*0.112*	*0.358*	*0.684*
**Financial hardship**								
Massive	Ref	Ref	Ref	Ref	Ref	Ref	Ref	Ref
Some	**1.09 (0.65–1.82)**	1.49 (0.87–2.58)	**0.66 (0.39–1.11)**	0.81 (0.47–1.39)	**1.50 (0.90–2.49)**	1.78 (1.04–3.02)	**0.55 (0.31–0.98)**	0.69 (0.38–1.24)
None	**0.67 (0.43–1.05)**	1.05 (0.64–1.73)	**0.42 (0.27–0.67)**	0.61 (0.37–1.01)	**0.99 (0.64–1.52)**	1.33 (0.83–2.12)	**0.51 (0.32–0.81)**	0.73 (0.44–1.21)
*p-value*	*0.004*	*0.111*	< *0.001*	*0.063*	*0.041*	*0.084*	*0.020*	*0.361*
**Supplementary benefits**								
Yes	Ref	Ref	Ref	Ref	Ref	Ref	Ref	Ref
No	0.70 (0.45–1.06)	0.83 (0.53–1.30)	**0.45 (0.29–0.71)**	**0.55 (0.35–0.88)**	1.19 (0.79–1.79)	1.37 (0.89–2.11)	**0.57 (0.36–0.91)**	0.67 (0.41–1.08)
*p-value*	*0.095*	*0.413*	< *0.001*	*0.011*	*0.413*	*0.153*	*0.018*	*0.100*
**Subjective social status**								
Range 1–10	**0.89 (0.83–0.94)**	0.95 (0.88–1.01)	**0.94 (0.88–0.99)**	0.98 (0.92–1.05)	0.99 (0.93–1.05)	1.01 (0.95–1.08)	0.93 (0.87–1.00)	0.98 (0.91–1.06)
*p-value*	< *0.001*	*0.119*	*0.031*	*0.610*	*0.715*	*0.774*	*0.063*	*0.662*

Results based on imputed data (*n* = 1,294). Missing values in % of total sample for inpatient stays: 3.6%; outpatient clinic visits: 3.3%; specialized SCI-centers: 5.5%; emergency departments: 3.3%. **Results in bold** indicate statistically significant associations (*p* < 0.05)

Models 1: unadjusted; Models 2: adjusted for age, gender, SCI severity, time since injury, driving time to closest SCI-center, secondary health conditions, mental health, and comorbidities

*Abbreviations: Ref* Reference group, *SCI* Spinal Cord Injury

## Discussion

This study provides initial evidence for social inequalities in the utilization of some specific health providers and institutions within a population of people with physical impairments due to SCI in Switzerland. However, we generally find that the basic health care provision is guaranteed in this wealthy country, and that social inequalities were only observed for specific care providers. Most prominent social inequalities were found for the visits to specialists, dentists, and dental hygienists, with persons from higher SES groups reporting higher likelihood for having visited those providers in the past 12 months. Moreover, we observed that persons with higher education reported more visits to pharmacists, persons with higher income more visits to natural healers, and persons with higher subjective social status more visits to chiropractors. Also, persons granted supplementary benefits were more likely to visit general practitioners and receiving support from home care services and persons with higher SES tended to visit a larger number of different health care providers. In contrast, we found statistically non-significant trends towards lower likelihood for inpatient stays, outpatient clinic, and emergency department visits and enhanced likelihood to visit a specialized SCI-center in higher SES groups. Effects of the perceived financial hardship on health service utilization was more volatile and mostly statistically insignificant. Although this study did not detect pronounced social inequalities other than in the utilization of specialists and dental care providers, findings nevertheless highlight the importance of including different SES indicators in research that aims to identify drivers of inequalities in health service utilization in people with SCI, as different SES dimensions relate to different resources important for health service utilization. SES-indicator specific findings are discussed and interpreted in the following paragraph.

In line with previous studies [3–5, 7, 32], we found an increased likelihood for specialist visits in higher SES groups. Earlier findings suggested that increased awareness for the importance of own health, enhanced cultural capital and larger social networks leading to better navigation in the complex health system, and differences in information policies of general practitioners might contribute to the inequalities in specialist visits [7]. Increased awareness and higher health literacy in higher SES groups [33, 34], might present the critical arguments for the utilization of yearly routine medical check-ups, which are often performed by specialists in Switzerland and thus explain enhances specialist visits. Also, previous studies support the notion that preventive screenings are more prevalent in higher SES groups [35, 36]. Our finding that higher SES groups report higher odds for dentists and dental hygienist visits confirms a well-known phenomenon [11, 12, 37, 38]. Besides the fact that visits to dentists and dental hygienists are usually not covered by health insurance and thus reinforcing income inequalities in utilization, similar arguments can be used to explain the differences in utilization as other preventive medical treatments. It is however important to mention that the costs for dental care of persons with supplementary benefits are covered by the health insurance. The lower likelihood to visit dental hygienists can thus not be explained by the scarcity of financial resources, but is probably due to other factors, such as limited awareness for the importance of oral health or difficulties in overcoming environmental barriers (e.g., lack of transportation, no wheelchair accessible dental surgery). Further, it might even be the case that persons with supplementary benefits are not aware that the costs are overtaken by the insurance, or that bureaucratic barriers in the reimbursement of costs hinder people in utilizing this service. Persons with SCI are particularly vulnerable to poor oral health and poor oral health can have drastic effects on the immune system or the development of pressure ulcers [39, 40]. Therefore, specific interventions to enhance the utilization of oral health care services in lower SES groups are highly recommended.

The result showing that persons with higher education and higher subjective social status had higher odds to visit pharmacists is difficult to interpret. As many general practitioners in Switzerland are authorized to dispense medications, these findings possibly mirror the fact that persons from lower educational or subjective status groups more often obtain medications directly from general practitioners, and therefore do not need to visit pharmacists. This assumption is supported by the fact that we observed a slight tendency for more general practitioner visits in persons with lower education and lower subjective status. Further, the finding that persons with higher income reported a higher likelihood to visit natural healers might directly relate to financing issues. In Switzerland, only a selection of officially acknowledged alternative medical treatments (e.g., traditional Chinese medicine, homeopathy) are compensated by health insurances and any alternative treatment apart from these recognized treatments must be payed out of pocket, thus potentially limiting access from lower income groups. The trend that persons granted supplementary benefits were more likely to visit general practitioners has been observed for the general Swiss population [10] and might be explained by the fact that persons with supplementary benefits more often live in institutions, where basic health care is provided by the general practitioners. Moreover, elderly persons are at higher risk to be granted supplementary benefits, and elderly persons more often report long-term care relationships with general practitioners than younger persons [41]. The higher support from home care services in persons receiving supplementary benefits might be explained by financial reasons, as health insurance fully covers the cost of home care for persons who are granted supplementary benefits, whereas others usually have to contribute a considerable part of the financing for home care services by themselves. It might also be the case that persons without supplementary benefits can more often rely on informal caregiving from family members and are thus less dependent on formal home care.

It is finally highly likely that persons in higher SES groups generally have more financial and knowledge-related resources to navigate the complex health system and use a combination of different treatments, as represented in the findings of higher number of different health care providers visited in higher SES groups. In contrast, our study did not provide evidence for social inequalities in the utilization of different health care institutions, as results on associations between any SES indicator and inpatient stays, visits to outpatient clinics, emergency departments and specialized SCI-centers were insignificant. Although we found non-significant trends showing slightly increased likelihood for higher emergency department visits in lower SES groups, previous general population findings [32] were not replicated in our sample of persons with SCI in Switzerland. This suggests that primary care provision only marginally follows social patterns and that access to basic health care is guaranteed for all persons with SCI, preventing persons from lower SES groups of high utilization of emergency departments. One reason for the lack of social inequalities in the utilization of those institutions might be that persons with SCI usually undergo long initial rehabilitation programs, where they are specifically educated in health management, irrespective of their socioeconomic conditions.

### Strengths and limitations

The SwiSCI community survey provides a large population-based data base with a well-defined sampling frame and neglectable response bias related to known sociodemographic and lesion characteristics [18]. Moreover, we adjusted final models for health states, allowing an understanding of SES-patterns in health service utilization that is not due to the different health care needs, but aims to understand ‘true’ differences between different SES groups. However, given that the SES of non-respondents was not assessed, we cannot evaluate whether we adequately included the most deprived groups or whether there is a selection bias towards over-representation of persons from higher SES groups. Results might be more pronounced if particularly disadvantaged individuals in lower SES groups, such as persons with low SES and language barriers, would have been included as well. It remains further unknown whether we missed some relevant, unmeasured confounders that impact on SES as well as on health service utilization. Also, other dimensions of potential inequalities in health service utilization (e.g. gender, race, ethnicity) were not included in this study and potential mediating path through which SES impacts on service utilization (e.g. health literacy) were not assessed. Moreover, the cross-sectional nature of the data prevents inferences about causal relationships. However, it seems reasonable to assume that SES indicators affect the health service utilization, and not vice versa. Also, the use of self-report data on the reporting of visits to the health care providers and institutions during the past 12 months might be subject to recall bias. Furthermore, the high amount of missing values in some of the constructs (e.g. household income) might limit the robustness of comparisons between analyses based on full case vs. imputed data.

## Conclusions

Given that we did not observe pronounced social inequalities in most of the included indicators for health service utilization, this study provides evidence that the basic health care provision is guaranteed for all patients with SCI in Switzerland, independently of their SES. However, social inequalities were still observed for the utilization of specific providers, such as oral health care providers. As persons with SCI are vulnerable to dental health problems and that good dental health is key for health maintenance, specific interventions to enhance regular dental check-ups in lower SES groups are highly recommended.

## Supplementary Information

**Additional file 1: **Unadjusted associations between indicators of the socioeconomic status and visits to specific health care providers: Odds ratio (OR) and 95% confidence intervals (CI) for the likelihood to visit specific providers and coefficients (coeff) and 95% CI for the total number of different providers visited during the past 12 months by indicators of the socioeconomic status.

## Data Availability

The datasets used and/or analysed during the current study are available from the corresponding author on reasonable request.

## Abbreviations

CI: Confidence interval
IQR: Interquartile range
MHI-5: SF-36 5-item Mental Health Index
MI: Multiple imputation
OR: Odds ratio
OECD: Organization for Economic Co-operation and Development
SCI: Spinal cord injury
SCI-SCS: Spinal Cord Injury Secondary Conditions Scale
SD: Standard deviation
SES: Socioeconomic status
SwiSCI: Swiss Spinal Cord Injury Cohort Study
