# Supplementary material for: Does the socioeconomic status predict health service utilization in persons with enhanced health care needs? Results from a population-based survey in persons with spinal cord lesions from Switzerland
Source: Int J Equity Health. 2022 Jul 12;21:94. doi: 10.1186/s12939-022-01693-6 (PMC9275068; doi:10.1186/s12939-022-01693-6)
Supplement: Supplementary file 1 — Additional file 1: Unadjusted associations between indicators of the socioeconomic status and visits to specific health care providers: Odds ratio (OR) and 95% confidence intervals (CI) for the likelihood to visit specific providers and coefficients (coeff) and 95% CI for the total number of different providers visited during the past 12 months by indicators of the socioeconomic status. [file 12939_2022_1693_MOESM1_ESM.docx]

**Appendix 1.** Unadjusted associations between indicators of the socioeconomic status and visits to specific health care providers: Odds ratio (OR) and 95% confidence intervals (CI) for the likelihood to visit specific providers and coefficients (coeff) and 95% CI for the total number of different providers visited during the past 12 months by indicators of the socioeconomic status

|  | **General practitioner** | **SCI- specialist** | **Other specialists** | **Dentist** | **Dental hygienist** | **Physiotherapist** | **Occupational therapist** | **Chiropractor** | **Home care service** | **Masseur** | **Pharmacist** | **Natural healer** | **Psychologist** | **N° of different health care providers visited (range 0-13)** |
| --- | --- | --- | --- | --- | --- | --- | --- | --- | --- | --- | --- | --- | --- | --- |
| Persons using service | N (%) | N (%) | N (%) | N (%) | N (%) | N (%) | N (%) | N (%) | N (%) | N (%) | N (%) | N (%) | N (%) | Mean (95% CI) |
|  | 1088 (84.1) | 544 (42.0) | 673 (52.0) | 823 (63.6) | 657 (50.8) | 833 (64.4) | 215 (16.6) | 69 (5.3) | 281 (21.7) | 245 (18.9) | 498 (38.5) | 178 (13.7) | 111 (8.6) | 4.80 (4.69-4.92) |
|  | OR (95% CI) | OR (95% CI) | OR (95% CI) | OR (95% CI) | OR (95% CI) | OR (95% CI) | OR (95% CI) | OR (95% CI) | OR (95% CI) | OR (95% CI) | OR (95% CI) | OR (95% CI) | OR (95% CI) | Coeff (95% CI) |
| **Education** |  |  |  |  |  |  |  |  |  |  |  |  |  |  |
| Compulsory | Ref | Ref | Ref | Ref | Ref | Ref | Ref | Ref | Ref | Ref | Ref | Ref | Ref | Ref |
| Higher secondary | 0.90 (0.57-1.42) | **1.25 (0.90-1.73)** | **1.12 (0.81-1.53)** | 1.09 (0.78-1.52) | **1.89 (1.36-2.62)** | 1.08 (0.78-1.51) | 1.10 (0.72-1.68) | 1.39 (0.59-3.25) | 0.88 (0.61-1.27) | 1.31 (0.85-2.03) | **1.22 (0.88-1.71)** | 1.13 (0.69-1.85) | 0.82 (0.47-1.43) | **0.34 (0.01-0.68)** |
| Short tertiary | 1.21 (0.68-2.16) | **1.48 (1.01-2.18)** | **1.30 (0.89-1.89)** | 1.36 (0.92-2.02) | **1.54 (1.73-3.74)** | 0.99 (0.68-1.46) | 0.98 (0.59-1.62) | 2.48 (1.00-6.13) | 0.77 (0.49-1.21) | 2.10 (1.29-3.41) | **1.22 (0.82-1.81)** | 1.57 (0.91-2.71) | 0.97 (0.51-1.86) | **0.69 (0.28-1.09)** |
| Tertiary | 0.61 (0.37-1.00) | **1.83 (1.26-2.66)** | **1.62 (1.12-2.34)** | 1.41 (0.96-2.09) | **2.92 (2.01-4.26)** | 1.15 (0.78-1.69) | 0.93 (0.56-1.54) | 1.93 (0.76-4.89) | 0.77 (0.49-1.19) | 1.37 (0.84-2.24) | **1.86 (1.28-2.71)** | 1.56 (0.91-2.67) | 1.16 (0.63-2.15) | **0.80 (0.41-1.18)** |
| *p-value* | *0.074* | *0.005* | *0.022* | *0.177* | *<0.001* | *0.775* | *0.697* | *0.348* | *0.5003* | *0.397* | *0.003* | *0.195* | *0.392* | *<0.001* |
| **Household income** |  |  |  |  |  |  |  |  |  |  |  |  |  |  |
| Income deciles 1-10 | 0.95 (0.90-1.01) | 1.05 (1.00-1.10) | 1.01 (0.96-1.05) | **1.05 (1.00-1.10)** | **1.13 (1.08-1.19)** | 0.98 (0.94-1.03) | 0.99 (0.93-1.06) | 1.02 (0.92-1.13) | 0.99 (0.93-1.04) | 1.03 (0.97-1.08) | 1.01 (0.97-1.06) | 1.05 (0.99-1.12) | 0.96 (0.89-1.04) | **0.05 (0.00-0.98)** |
| *p-value* | *0.103* | *0.059* | *0.751* | *0.036* | *<0.001* | *0.388* | *0.811* | *0.684* | *0.626* | *0.369* | *0.567* | *0.127* | *0.343* | *0.033* |
| **Financial hardship** |  |  |  |  |  |  |  |  |  |  |  |  |  |  |
| Massive | Ref | Ref | Ref | Ref | Ref | Ref | Ref | Ref | Ref | Ref | Ref | Ref | Ref | Ref |
| Some | 1.34 (0.69-2.64) | 0.98 (0.60-1.61) | **0.64 (0.38-1.06)** | 1.07 (0.65-1.78) | **1.54 (0.92-2.57)** | 1.16 (0.69-1.96) | **1.17 (0.64-2.14)** | 1.01 (0.31-3.33) | 1.26 (0.69-2.28) | 0.85 (0.47-1.54) | 0.78 (0.47-1.29) | 0.89 (0.42-1.86) | **0.41 (0.20-0.84)** | -0.06 (-0.59-0.48) |
| None | 1.16 (0.66-2.05) | 0.83 (0.54-1.27) | **0.57 (0.37-0.89)** | 1.43 (0.92-2.22) | **2.14 (1.37-3.45)** | 1.01 (0.65-1.58) | **0.65 (0.37-1.12)** | 1.05 (0.37-2.95) | 0.92 (0.54-1.56) | 0.69 (0.41-1.14) | 0.75 (0.49-1.16) | 1.01 (0.53-1.91) | **0.33 (0.19-0.58)** | -0.21 (-0.67-0.24) |
| *p-value* | *0.669* | *0.431* | *0.046* | *0.075* | *<0.001* | *0.694* | *0.004* | *0.993* | *0.218* | *0.218* | *0.436* | *0.857* | *<0.001* | *0.458* |
| **Supplementary benefits** |  |  |  |  |  |  |  |  |  |  |  |  |  |  |
| Yes | Ref | Ref | Ref | Ref | Ref | Ref | Ref | Ref | Ref | Ref | Ref | Ref | Ref | Ref |
| No | **0.31 (0.13-0.72)** | 1.42 (0.93-2.17) | 0.71 (0.47-1.08) | 1.02 (0.66-1.55) | **1.59 (1.05-2.41)** | 0.94(0.61-1.45) | 0.93 (0.54-1.58) | 1.81 (0.56-5.88) | **0.56 (0.36-0.88)** | 1.06 (0.62-1.80) | 0.79 (0.52-1.20) | 1.48 (0.76-2.91) | **0.46 (0.26-0.82)** | -0.19 (-0.62-0.25) |
| *p-value* | *0.007* | *0.109* | *0.109* | *0.947* | *0.029* | *0.791* | *0.777* | *0.325* | *0.011* | *0.831* | *0.270* | *0.250* | *0.008* | *0.398* |
| **Subjective social status** |  |  |  |  |  |  |  |  |  |  |  |  |  |  |
| Range 1-10 | **0.90 (0.83-0.98)** | 1.05 (0.99-1.12) | 0.96 (0.90-1.01) | **1.10 (1.04-1.17)** | **1.18 (1.11-1.25)** | 0.96 (0.91-1.02) | **0.89 (0.83-0.97)** | **1.16 (1.01-1.32)** | 0.95 (0.89-1.02) | 1.06 (0.98-1.14) | 1.03 (0.97-1.10) | 1.06 (0.97-1.15) | **0.88 (0.79-0.97)** | 0.04 (-0.03-0.10) |
| *p-value* | *0.013* | *0.075* | *0.140* | *0.002* | *<0.001* | *0.222* | *0.005* | *0.033* | *0.156* | *0.136* | *0.259* | *0.213* | *0.012* | *0.242* |

The overall missing values for different health services use: n=27 (2.1%). Results based on imputed data (n=1,294). *Abbreviations:* Ref: Reference group; SCI: Spinal cord injury. **Results in bold** indicate statistically significant associations (p<0.05).
